# Brain‐derived neurotropic factor polymorphisms, traumatic stress, mild traumatic brain injury, and combat exposure contribute to postdeployment traumatic stress

**DOI:** 10.1002/brb3.392

**Published:** 2015-12-17

**Authors:** Michael N. Dretsch, Kathy Williams, Tanja Emmerich, Gogce Crynen, Ghania Ait‐Ghezala, Helena Chaytow, Venkat Mathura, Fiona C. Crawford, Grant L. Iverson

**Affiliations:** ^1^ U.S. Army Aeromedical Research Laboratory6901 Farrel RoadFort RuckerAlabama22206; ^2^ National Intrepid Center of ExcellenceWalter Reed National Military Medical Center4860 South Palmer RoadBethesdaMaryland20889; ^3^ Human Dimension Division (HDD)Headquarters Army Training and Doctrine Command (HQ TRADOC)950 Jefferson AveFort EustisVirginia23604; ^4^ Roskamp Institute2040 Whitfield AveSarasotaFlorida34243; ^5^ Department of Physical Medicine and RehabilitationHarvard Medical SchoolBostonMassachusetts; ^6^ Spaulding Rehabilitation HospitalBostonMassachusetts; ^7^ Red Sox Foundation and Massachusetts General Hospital Home Base ProgramBostonMassachusetts; ^8^ Defense and Veterans Brain Injury CenterBethesdaMaryland; ^9^ Center for Health and RehabilitationDepartment of Physical Medicine & RehabilitationHarvard Medical School79/96 Thirteenth StreetCharlestown Navy YardCharlestownMassachusetts02129

**Keywords:** BDNF, deployment, genetics, military, posttraumatic stress disorder, psychological health, traumatic brain injury

## Abstract

**Background:**

In addition to experiencing traumatic events while deployed in a combat environment, there are other factors that contribute to the development of posttraumatic stress disorder (PTSD) in military service members. This study explored the contribution of genetics, childhood environment, prior trauma, psychological, cognitive, and deployment factors to the development of traumatic stress following deployment.

**Methods:**

Both pre‐ and postdeployment data on 231 of 458 soldiers were analyzed. Postdeployment assessments occurred within 30 days from returning stateside and included a battery of psychological health, medical history, and demographic questionnaires; neurocognitive tests; and blood serum for the D2 dopamine receptor (DRD2), apolipoprotein E (APOE), and brain‐derived neurotropic factor (BDNF) genes.

**Results:**

Soldiers who screened positive for traumatic stress at postdeployment had significantly higher scores in depression (*d *=* *1.91), anxiety (*d *=* *1.61), poor sleep quality (*d *=* *0.92), postconcussion symptoms (*d *=* *2.21), alcohol use (*d *=* *0.63), traumatic life events (*d *=* *0.42), and combat exposure (*d *=* *0.91). BDNF Val66 Met genotype was significantly associated with risk for sustaining a mild traumatic brain injury (mTBI) and screening positive for traumatic stress. Predeployment traumatic stress, greater combat exposure and sustaining an mTBI while deployed, and the BDNF Met/Met genotype accounted for 22% of the variance of postdeployment PTSD scores (*R* ^*2*^ = 0.22, *P *<* *0.001). However, predeployment traumatic stress, alone, accounted for 17% of the postdeployment PTSD scores.

**Conclusion:**

These findings suggest predeployment traumatic stress, genetic, and environmental factors have unique contributions to the development of combat‐related traumatic stress in military service members.

## Introduction

A substantial minority of service members screen positive for posttraumatic stress disorder (PTSD) following their deployment (Tanielian and Jaycox 2008). The Veterans’ Health Administration (VHA) has spent $2.2 billion on patients with PTSD between 2004 and 2009 (Congressional Budget Office, 2012). Recent predictions suggest these costs will increase over the next several years (2015 Congressional Submission). Service members who have a premilitary or predeployment history of traumatic events (Youngner et al. 2012), mental health problems (Spinhoven et al. 2014; Stander et al. 2014), and lower psychological resilience (Zerach et al. 2013) are at increased risk for having a traumatic stress condition such as PTSD. Civilian and military studies have also linked lower intelligence (Breslau et al. 2006) and lower scores on neuropsychological tests (Marx et al. 2009) to the future development of traumatic stress. During their deployment, service members who are exposed to greater combat intensity (Vasterling et al. 2010), sleep deprivation or insufficiency (Meerlo et al. 2008), those who suffer a mild traumatic brain injury (mTBI) (Yurgil et al. 2014), or sustain severe orthopedic or bodily injury (Vranceanu et al. 2014) are at increased risk for traumatic stress. Service members and veterans who have a traumatic stress condition often have other problems, such as depression (Rojas et al. 2014), chronic pain (Alschuler and Otis 2014), sleep problems (Vandrey et al. 2014), substance abuse (Rojas et al. 2014; Vandrey et al. 2014), and marital and family problems (Gerlock et al. 2013).

Less is known about how genetic factors influence or moderate risk for psychological injuries in service members or how genetics might be associated with outcomes following deployment to a war zone. It is generally accepted in psychiatry that many mental health problems arise from the cumulative effects of genetics, childhood adversity, and life stress (Uher 2014). Researchers have identified genetic factors that are associated with risk for traumatic stress disorder in adults, mostly through civilian studies.

The apolipoprotein E (APOE) gene has been linked to multiple neurological and neuropsychiatric conditions; the most often reported being Alzheimer's disease (Burke and Roses 1991; Seripa et al. 2011; Panza et al. 2012). APOE genotype has been implicated in hypothalamic–pituitary–adrenal (HPA) axis regulation and hippocampal and amygdala volume (Raber et al. 2000; O'Dwyer et al. 2012; Soldan et al. 2015). Outcome after TBI also is influenced by APOE genotype, in both humans and laboratory models, with risk for poor outcome in the order *ε*4 > *ε*3 > *ε*2. (D'Onofrio et al. 2011; Esiri and Chance 2012). The *ε*4 allele is linked to increased rates of PTSD in combat exposed Vietnam veterans, while the *ε*2 allele confers risk for combat‐related PTSD in Korean and Vietnam War veterans (Kim et al. 2013; Lyons et al. 2013).

The D2 dopamine receptor gene (DRD2) is inconsistently implicated in substance abuse, Alzheimer's disease, schizophrenia, and PTSD (Noble 2003). Some evidence suggests the A1 allele has protective properties for a range of mental health symptoms in Vietnam veterans (Lawford et al. 2006), and other studies report an increased risk conferred by the A1 allele in Vietnam veterans (Comings et al. 1996). Moreover, the single nucleotide polymorphism of the 957C > T in the DRD2 gene is a risk factor for PTSD and has been associated with depression, anxiety, and impaired social function (Voisey et al. 2009). The functional significance of the polymorphism is still unclear, although it has been associated with a 40% reduction in the expression of D2 receptors in the striatum without change in receptor affinity (Noble et al. 1991; Thompson et al. 1997; Pohjalainen et al. 1998; Jonsson et al. 1999; Wong et al. 2000; Ritchie and Noble 2003). Therefore, due to being implicated in combat‐related psychopathology in aging veterans, DRD2 is a good candidate to investigate the genetic contribution to PTSD traumatic stress conditions in active‐duty service members.

The brain‐derived neurotropic factor (BDNF) gene has also been implicated in several neuropsychiatric disorders (Zhang et al. 2006). BDNF has a role in activity‐dependent neural plasticity processes of the hippocampus and amygdala, implicated in long‐term learning and memory (Hariri et al. 2003; Andero and Ressler 2012; Mahan and Ressler 2012). In one study, low BDNF serum levels were associated with PTSD, while another reported no relationship to PTSD in victims of urban violence (Valente et al. 2011). However, the Met allele (one and two alleles) of BDNF Val66Met polymorphism may be associated with PTSD severity in veterans (Pivac et al. 2012).

This study prospectively explores the contribution of several genes (APOE, DRD2, and BDNF), predeployment traumatic events, predeployment mental health problems, neurocognitive functioning, combat exposure, early childhood environment, and sustaining an mTBI to the development of traumatic stress in active‐duty service members deployed to a combat environment. We hypothesized that predeployment mental health problems, combat exposure, and sustaining an mTBI in theater would be related to postdeployment traumatic stress. Because the findings to date regarding genetic contributions to PTSD in military service members and veterans are inconsistent and diverse, we did not have specific a priori genetic hypothesis.

## Materials and Methods

### Participants

Predeployment data from 458 U.S. Army soldiers who voluntarily participated in this study were collected within approximately 30 days prior to a 12 month deployment to the Middle East for Operation Iraqi Freedom (OIF)/Operation Enduring Freedom (OEF). There were no inclusion/exclusion criteria because all soldiers were deemed medically fit for deployment (i.e., physical and psychiatric screening) through the deployment medical screening. Due to attrition, data on only 231 of the 458 participants were also collected within 30 days of returning from their deployment (i.e., postdeployment). Attrition at postdeployment was due to inherent logistical difficulties when collecting data in different states; distance to testing facility at the military instillation, coordination of the team of specialized personnel necessary for collecting data, the sporadic return of soldiers (e.g., as individuals or small groups), coordination with brigade combat team leadership (e.g., last minute notification they had returned from deployment), participant withdrawal, and loss of participants due to injury and death. The demographics information for this group is provided in Table 1. The APOE, DRD2, and BDNF genotypes were determined for each participant (Table 2). One participant had missing data on the questionnaires, so some of the data analyses are presented for 230 participants.

**Table 1:** Demographic characteristics and predeployment test scores from those who completed the study versus were not seen following deployment

	Completed study (*N* = 230)	Not seen postdeployment (*N* = 227)
Age (*M*, SD)	25.3(6.4)	26.7(7.5)
Sex, male (%)	96.1%	91.6%
Education ≤12 years (%)	53.2%	51.1%
>12 years (%)	46.8%	48.9%
Race: White (%)	80.5%	72.7%
Black (%)	9.5%	10.6%
Hispanic/Latino (%)	6.1%	7.9%
Pacific/Islander (%)	1.3%	4.4%
Asian (%)	0.4%	0.4%
Native American (%)	0.4%	1.8%
Other (%)	1.7%	2.2%
Rank: Junior enlisted (%)	39.0%	34.8%
Noncommissioned officer (%)	58.9%	60.4%
Senior noncommissioned officer (%)	2.2%	3.5%
Past deployments: Zero	61.0%	54.6%
1 Prior deployment	23.4%	22.9%
2 Prior deployments	10.8%	16.2%
3 Prior deployments	3.9%	2.6%
4 or more prior deployments	0.8%	3.1%
PTSD Checklist—Military Version (*M*, SD)	21.3 (7.2)	23.5 (10.2)
Zung Depression Scale (*M*, SD)	33.9 (8.4)	35.4 (9.5)
Zung Anxiety Scale (*M*, SD)	31.7 (7.0)	31.2 (7.5)
Epworth Sleepiness scale (*M*, SD)	8.3 (4.4)	8.0 (4.1)
Pittsburgh Sleep Quality Index (*M*, SD)	6.2 (3.8)	6.1 (3.5)
Alcohol Use/Dependency Identification Test (*M*, SD)	6.1 (5.1)	6.8 (5.6)
CNS‐VS Neurocognitive Composite Index (*M*, SD)	92.5 (12.7)	93.6 (13.7)

PTSF, posttraumatic stress disorder; CNS‐VS, Central Nervous System‐Vital Signs^®^; M, Mean; SD; standard deviations.

**Table 2:** Comparison of APOE, BDNF, and DRD2 genotype distributions from those who completed the study versus were not seen following deployment

Genotype	Completed study (*N* = 231)		Not seen postdeployment (*N* = 227)	
	Frequency	Percentage	Frequency	Percentage
APOE				
E2/E2	2	0.9	1	0.4
E2/E3	20	8.7	30	13.2
E2/E4	5	2.2	4	1.8
E3/E3	156	67.5	147	64.8
E3/E4	47	20.3	43	18.9
E4/E4	1	0.4	2	0.9
BDNF				
Met/Met	12	5.1	6	2.6
Val/Met	57	24.7	76	33.5
Val/Val	157	68.4	142	62.6
Missing	4	1.7	3	1.3
DRD2				
A1/A1	12	5.2	17	7.5
A1/A2	82	35.5	77	33.9
A2/A2	136	58.9	132	58.1
Missing	1	0.4	1	0.4

APOE, apolipoprotein; BDNF, brain‐derived neurotropic factor; DRD2, D2 dopamine receptor gene.

### Procedures

Soldiers were given the opportunity to voluntarily participate after receiving a study brief by the PI at a designated facility on the military instillation. Data were collected from two brigade combat teams deploying to Iraq and Afghanistan. Informed consent was obtained for all participants after the nature of the study was explained with the presence of an ombudsperson. Consented participants were then escorted as a group to a separate classroom for computerized testing and/or the phlebotomy stations where several tubes of blood were drawn for genotyping and stored in a dry ice freezer prior to delivering to a −80°C medical specimen freezer at the end of each day. The procedures at pre‐ and postdeployment data collection phases were identical with the exception of a few additional deployment‐related measures at postdeployment. Participants received a check for $50 for each blood draw (pre‐ and postdeployment); thus, they could be compensated up to $100 for their participation.

Approval was attained from brigade commanders. The study was carried out in accordance with the latest version of the Declaration of Helsinki and the protocol and procedures were approved by the Institutional Review Board at Headquarters U.S. Army Medical Research and Materiel Command, Fort Detrick, MD.

### DNA genotyping

#### APOE

For amplification and digestion of the APOE gene from extracted DNA, we used a direct APOE kit (EzWay Direct APOE Genotyping Kit; Koma Biotechnology, Seoul, Korea), following manufacturer's instructions. Genotype‐specific fragments were separated by electrophoresis in a 3% metaphor agarose gel, stained with ethidium bromide.

#### DRD2

Using 0.125 *μ*L iTaq polymerase enzymes and 0.5 *μ*L DRD2 A1/A2‐specific primers (Eurofins, Luxembourg, Germany), 0.5 *μ*L extracted DNA per sample was amplified at the DRD2 region. Primers for DRD2 Val66Met were as follows: forward 5′–CCG TCG ACG GCT GGC CAA GTT GTC TA–3′ and reverse 5′–CCG TCG ACC CTT CCT GAG TGT CAT CA–3′. Reaction volume was 25 *μ*L with 0.75 *μ*L 50 mmol/L MgCl_2_, 0.5 *μ*L 10 mmol/L dNTP mix, and 2.5 *μ*L iTaq 10X buffer. PCR conditions were as follows: 5 min at 94°C, followed by 35 cycles for 1 min, 1 min, and 1.5 min at 94°C, 55°C, and 92°C, respectively. The PCR was terminated at 72°C for 10 min and held at 4°C. The amplified sequence was 310‐bp long (PCR not shown). The PCR products were digested with the 0.5 μL Taq I*α* enzyme (Biolabs, Ipswich, Massachusetts) for 16 h at 65C, which produced DNA fragments that correspond with A1 allele and A2 allele. Genotype‐specific fragments were separated by electrophoresis in a 3% metaphor agarose gel, stained with ethidium bromide.

#### BDNF

Using 0.125 *μ*L iTaq polymerase enzymes and 0.5 *μ*L BDNF‐specific primers (Eurofins), 0.5 *μ*L extracted DNA per sample was amplified at the BDNF region. Primers for BDNF Val66Met were as follows: forward 5′–AAA CAT CCG AGG ACA AGG TG–3′ and reverse 5′–ACG TGT ACA AGT CTG CGT CC–3′. Reaction volume was 25 *μ*L with 0.75 *μ*L 50 mmol/L MgCl_2_, 0.5 *μ*L 10 mmol/L dNTP mix, and 2.5 *μ*L iTaq 10X buffer. PCR conditions were as follows: 5 min at 94°C, followed by thirty 30 sec cycles of 94°C, 60°C, and 72°C. The PCR was terminated at 72°C for 10 min and held at 4°C. The product of this amplification was digested with 1 *μ*L Pml I enzyme (Biolabs) at 37°C for 16 h into genotype‐specific fragments, which were then separated by electrophoresis in a 3% metaphor agarose gel, stained with ethidium bromide.

### Psychological tests and questionnaires

#### PTSD Checklist—Military Version (Weathers et al. 1991)

The PTSD Checklist—Military Version (PCL‐M) is a 17‐item self‐report measure of the *Diagnostic and Statistical Manual of Mental Disorders—Fourth Edition* (*DSM‐IV*) symptoms of PTSD. Suggested cut‐point scores ≥30 have the best sensitivity and specificity for screening active‐duty military service members for traumatic stress (Bliese et al. 2008).

#### Life Events Checklist

The Life Events Checklist (LEC) (Gray et al. 2004) is a 17‐item self‐report measure designed to screen for potentially traumatic events in a respondent's lifetime. The LEC assesses exposure to 16 events known to potentially result in PTSD or distress and includes one item assessing any other extraordinarily stressful event not captured in the first 16 items.

#### Combat Exposure Scale

The Combat Exposure Scale (CES) (Guyker et al. 2013) is a 7‐item self‐report measure that assesses wartime stressors experienced by combatants. Items are rated on a 5‐point weighted scale on frequency and severity of exposure to a combat environment, physical engagement with the enemy, proximity to serious injury, and exposure to death.

#### Childhood Family Environment (also known as “childhood experiences” [CE]; King et al. 2006)

The CE is one of 13 subscales from the Deployment Risk and Resilience Inventory. The CE contains 15 items that measure different aspects of family dynamics during childhood. Lower scores indicate greater cohesion, accord, and closeness among family members.

#### Brief Traumatic Brain Injury Screen (BTBIS; Schwab et al. 2006)

This 3‐item assessment is used by the Army for identifying soldiers that may have sustained a mild TBI. Subjects identify the mechanism (e.g., fragment, vehicular, blast, etc.), symptoms immediately after the injury, and current symptoms believed to be associated with injury. An mTBI‐positive score required both the endorsement of an injury‐related event and at minimum, an altered state of consciousness (e.g., being dazed, confused, or seeing stars; or posttraumatic amnesia; loss of consciousness <20 min).

#### Neurobehavioral Symptom Inventory (Cicerone and Kalmar 1995)

The Neurobehavioral Symptom Inventory (NSI) is a 22‐item measure designed to evaluate self‐reported postconcussion symptoms (e.g., headache, fatigue, sensitivity to noise, sadness, difficulty concentrating, difficulty remembering, and visual problems). The scale yields three factor scores: physical, cognitive, and emotional. It is widely used in studies with active‐duty service members and veterans.

#### Zung Depression Scale (Zung 1965)

The Zung Depression Scale (ZDS) is a 20‐item self‐report depression rating scale. The ZDS produces scores ranging from 20 through 80; 20–44 for normal range, 45–59 for mildly depressed, 60–69 for moderately depressed, and 70 and above for severely depressed.

#### Zung Anxiety Scale (Zung 1971)

This Zung Anxiety Scale (ZAS) is a 20‐item self‐report anxiety rating scale. The ZAS has scores ranging from 20 through 80; 20–44 for normal range, 45–59 for mild‐to‐moderate anxiety, 60–74 for marked to severe anxiety, and 75–80 for extreme anxiety.

#### Alcohol Use Dependency Identification Test (AUDIT; Saunders et al. 1993)

This 10‐item instrument is used to assess alcohol use. A score of 8 or more in men, and 7 or more in women, indicates possible hazardous or harmful alcohol use. A score of 20 or more suggests potential alcohol dependence.

#### Epworth Sleepiness Scale (ESS; Johns 1991)

This instrument asks the subject to rate his or her probability of falling asleep on a scale of increasing probability from 0 to 3 in eight different situations.

#### Pittsburgh Sleep Quality Index (Buysse et al. 1989)

This 19‐item instrument is used to measure sleep quality during the previous month and discriminates between good and poor sleepers. The Pittsburgh Sleep Quality Index (PSQI) assess several domains including subjective sleep quality, sleep latency, sleep duration, habitual sleep efficiency, sleep disturbances, use of sleep medications, and daytime dysfunction.

### Neurocognitive measures

Central Nervous System‐Vital Signs^®^ (CNS‐VS) is a computerized neurocognitive assessment battery (Gualtieri and Johnson 2006). The present study used five CNS‐VS subtests (verbal memory, symbol digit coding, Stroop test, continuous performance test, and shifting attention test). The CNS‐VS domain scores calculated were verbal memory (VM), complex attention (CA), reaction time (RT), processing speed (PS), cognitive flexibility (CF), and executive functioning (EF). Domain scores have a mean of 100 and standard deviation of 15. Domain scores were averaged to form a single score or Neurocognitive Composite Index (NCI).

### Statistical analyses

Analyses were conducted using SPSS 19.0. Mean (*M*) scores, standard deviations (SD), delta change scores (*Δ*), percentage change scores (%*Δ*), and effect size (ES) using Morris and DeShon (2002) equation for within‐subjects analysis (Cohen's *d*), which corrects for dependence between means. Chi‐square (*χ* ^2^) was used with categorical data. Wilcoxon signed‐rank test was used for assessing the significance of pre‐ and postdeployment changes in scores on the various psychological health measures. Multiple linear regression (backward extraction) was used to explore the contribution of various predeployment and deployment factors on postdeployment traumatic stress. Variables were tested for linearity, normality, heteroskedasticity, multicollinearity, and were log‐10 transformed when necessary. For independent variables, categorical data were dummy coded and continuous data were grand mean centered. Tables report only nontransformed variable data.

## Results

### Genotype

**Figure 1:**
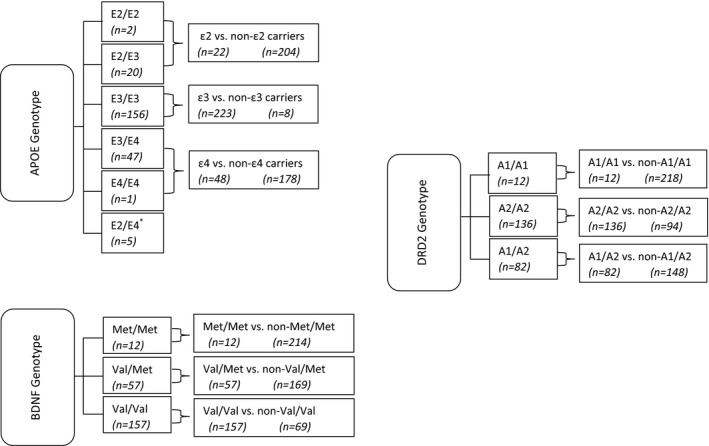
Allele distributions and grouping of the APOE, BDNF, and DRD2 genotypes for the postdeployment sample. *Not included in the analysis of ϵ2 vs. non‐ϵ2 and ϵ4 vs. non‐ϵ4 carriers.

The allele distributions and grouping of the APOE, BDNF, and DRD2 genotypes for the postdeployment sample are presented in Figure 1. There was no frequency violation of Hardy–Weinberg assumptions. To explore the contribution of specific genotypes, data were aggregated and dummy coded into subgroups based on allele carrier status.

### Psychological health outcomes and postdeployment traumatic stress

Pre‐ and postdeployment outcome measures for the total sample are compared in Table 3 (*N* = 230; one participant with incomplete data). Outcome measures stratified by traumatic stress status also are presented. Compared to participants that screened negative for traumatic stress (*n *=* *189; _post_PCL‐M < 30), participants positive for traumatic stress (*n *=* *41) had significantly higher scores in depression (*d *=* *1.91), anxiety (*d *=* *1.60), sleepiness (*d *=* *0.67), postconcussive symptoms (*d *=* *2.21), poor sleep quality (*d *=* *0.92), alcohol use (*d *=* *0.63), combat exposure (*d *=* *0.91), and prior traumatic events (*d *=* *0.42) (all *P* values <0.005, Bonferroni corrected *P*‐value). There were no significant differences in childhood environment (*d *=* *0.30) and the Neurocognitive Composite Index (*d *=* *0.40), after controlling for multiple comparisons (*P *>* *0.005, Bonferroni corrected *P*‐value).

**Table 3:** Mean scores (SD) of participants with both pre‐ and postdeployment data

	Predeployment (*N* = 230)	Postdeployment (*N* = 230)	Cohen's *d*	Postdeployment		
				No traumatic stress (*n* = 189)	Traumatic stress (*n* = 41)	Cohen's *d*
PTSD Checklist—Military Version	21.3 (7.2)	23.8 (8.3)	0.31	20.7 (3.9)	38.1 (8.6)	3.67
Zung Depression Scale	33.9 (8.4)	34.5 (7.9)	0.07	32.3 (6.4)	44.4 (6.3)	1.91
Zung Anxiety Scale	31.7 (7.0)	32.2 (6.5)	0.07	30.6 (5.4)	39.5 (6.2)	1.60
Epworth Sleepiness Scale	8.3 (4.4)	9.1 (4.0)	0.19	8.7 (3.9)	11.2 (4.1)	0.67
Pittsburgh Sleep Quality Index	6.2 (3.8)	7.3 (3.8)	0.23	6.7 (3.5)	10.0 (4.1)	0.92
Alcohol Use/Dependency Identification Test	6.1 (5.1)	5.4 (5.5)	0.32	4.8 (5.0)	8.2 (7.0)	0.62
CNS‐VS Neurocognitive Composite Index	92.5 (12.7)	95.4 (13.6)	0.22	96.6 (13.2)	91.4 (11.8)	0.40
Neurobehavioral Symptom Inventory	n/a	n/a	–	7.8 (8.1)	28.2 (14.3)	2.21
Childhood Experiences	55.0 (10.7)	n/a	–	55.5 (10.5)^1^	52.3 (11.4)^1^	0.30
Life Events Checklist	48.3 (16.4)	n/a	–	19.5 (16.6)^1^	26.3 (14.4)^1^	0.42
Combat Exposure Scale	n/a	10.0 (6.9)	–	8.9 (5.9)	14.8 (9.0)	0.91
mTBI, frequency	n/a	24 (10.6%)	–	15 (7.9%)	9 (22.0%)	–

^1^Completed prior to their deployment.

### Predictors of postdeployment traumatic stress

A multiple linear regression analysis (backward extraction method) was conducted with _post_PCL‐M scores as the dependent variable, and genotype (BDNF, APOE, and DRD2), predeployment cognitive functioning (_pre_NCI), combat exposure (CES), predeployment traumatic stress (_pre_PCL‐M), prior deployments, age, sex, race, sustaining an mTBI while deployed, childhood environment (CE), life events (LEC), and a CE by LEC interaction variable as the independent variables. The overall model was significant, adjusted *R* ^2^ = 0.24, *F*_18,201_ = 4.49, SE = 0.11, *P *<* *0.001, and accounted for 22% of the variability in _post_PCL‐M scores. Prior deployments, life events, cognitive functioning, age, race, sex, childhood environment, and APOE and DRD2 genotypes did not independently account for the variance in _post_PCL‐M scores, *P* > 0.05. As observed in Table 3, being a BDNF Met/Met carrier (*β *= 0.08, *t *=* *2.25, *P *=* *0.025), sustaining an mTBI (*β *= 0.05, *t *=* *1.99, *P *=* *0.048), higher levels of combat exposure (*β* = 0.07, *t *=* *3.43, *P *=* *0.001), and predeployment traumatic stress (*β *= 0.37, *t *=* *5.34, *P *<* *0.001) were associated with greater _post_PCL‐M scores. Importantly, _pre_PCL‐M alone accounted for 17% of the variance of the _post_PCL‐M, adjusted *R* ^2^ = 0.17, *F*_1,223_ = 45.33, *P *<* *0.001, followed by 8% for combat exposure, 4% for mTBI, and 3% for Met/Met. The same variables were significant after rerunning the regression analysis using a forced‐entry approach (Table 4).

**Table 4:** Results of multiple linear regression for independent predictors of postdeployment traumatic stress

Model variables	Unstandardized coefficients		Standardized coefficients	*t*	Sig.	95% Confidence interval	
	B	Std. Error	*β*			Lower bound	Upper bound
(Constant)	1.04	0.18	–	5.83	0.000	0.69	1.40
Met/Met	0.08	0.04	0.14	2.25	0.025	0.01	0.15
Combat Exposure Scale	0.07	0.02	0.21	3.43	0.001	0.03	0.11
_pre_PCL‐M	0.37	0.07	0.33	5.34	0.000	0.23	0.50
mTBI	0.05	0.03	0.12	1.99	0.048	0.01	0.10

### BDNF and traumatic stress

Because the BDNF Val66Met polymorphism was an independent significant predictor for deployment‐related traumatic stress, the frequency and percentage of various polymorphisms for participants who screened positive for traumatic stress (_post_PCL‐M ≥ 30) were calculated (see Table 5). The prevalence of screening positive for traumatic stress at postdeployment was 18.1% (*n *=* *41). The frequency of the Met/Met genotype participants who screened positive for PTSD (12.2%) was significantly greater compared to the no PTSD group (3.8%; *χ* ^2^(1) = 4.72, *P *= 0.030). There were no significant differences in the frequencies of Val/Val or Met/Val, or the other genotypes (i.e., APOE and DRD2), between participants with or without traumatic stress, *P *>* *0.05. Met/Met genotype carriers screened positive for traumatic stress 42% of the time, which was 2.97 times (*χ* ^2^(1) = 4.95, *P *=* *0.026; RR = 2.97, 95% CI [0.93, 7.69]) and 2.34 times (*χ* ^2^(1) = 4.03, *P *=* *0.045; RR = 2.34, 95% CI [0.86, 4.49]) more likely than Val/Met and Val/Val genotype carriers, respectively.

**Table 5:** APOE, BDNF, and DRD2 genotype frequencies stratified by postdeployment traumatic stress

Genotype	PTSD (*n* = 41)	No PTSD (*n* = 189)
APOE		
E2/E2	2 (4.9%)	0
E2/E3	4 (9.8%)	16 (8.5%)
E2/E4	1 (2.4%)	4 (2.1%)
E3/E3	25 (61.0%)	131 (69.3%)
E3/E4	8 (19.5%)	38 (20.1%)
E4/E4	1 (2.4%)	0
BDNF		
Val/Met	8 (19.6%)	49 (25.9%)
Val/Val	28 (68.3%)	129 (68.3%)
Met/Met	5 (12.2%)	7 (3.7%)
Missing	0	4 (2.1%)
DRD2		
A1/A1	2 (4.9%)	10 (5.3%)
A1/A2	14 (34.1%)	68 (36.0%)
A2/A2	25 (61.0%)	110 (58.2%)
Missing	0	1 (4.2%)

APOE, apolipoprotein; BDNF, brain‐derived neurotropic factor; DRD2, D2 dopamine receptor gene.

The percentage of the total sample that screened positively for traumatic stress was 17.8%. The percentages within genotypes that screened positively for traumatic stress were as follows: E2/E3 = 20.0%, E3/E3 = 16.0%, E3/E4 = 17.4%, Val/Met = 14.0%, Val/Val = 17.8%, Met/Met = 41.7%, A1/A1 = 16.7%, A1/A2 = 17.1%, and A2/A2 = 18.5% (only those genotypes with more than 10 subjects were included).

### BDNF, mild TBI, and PTSD

The frequency of the Met/Met genotype in participants who sustained an mTBI (4 of 24; 16.7%) while deployed was significantly greater than those who did not (9 of 203, 4.4%; *χ* ^2^(1) = 5.95, *P *=* *0.015). There were no significant differences in the frequencies of the Val/Val or Met/Val, or the other genotypes (i.e., APOE and DRD2), between participants with or without mTBI, *P *>* *0.05.

Participants who sustained an mTBI (*n *=* *24; 10.4%) during their deployment had a higher frequency of traumatic stress (9 of 24; 37.5%) compared to those that did not sustain an mTBI (32 of 206, 15.5%; *χ* ^2^(1) = 7.08, *P *=* *0.008. In addition, the mTBI group reported having significantly greater combat exposure (*M *=* *13.1, *SD* = 7.0) while deployed than those who did not sustain an mTBI (*M *=* *9.6, SD = 6.8; *U *=* *1,700.0, *Z *=* *−2.51, *P *=* *0.012, *d *=* *0.51, medium effect size), potentially suggesting a three‐way interaction between mTBI, combat exposure, and traumatic stress.

## Discussion

Soldiers who screened positively for traumatic stress following deployment reported greater predeployment traumatic life events and postdeployment depression, anxiety, sleep difficulty, problematic alcohol use, and postconcussive symptoms. In multivariate analyses, predeployment traumatic stress scores, BDNF Met/Met genotype, sustaining an mTBI while deployed, and exposure to combat events were all significant independent predictors of postdeployment traumatic stress. The BDNF Met/Met genotype was associated with a significantly greater proportion of soldiers with traumatic stress. Only 5.2% of the total sample had the BDNF Met/Met genotype. Of those, however, 42% screened positively for traumatic stress. The BDNF Met/Met genotype was also associated with increased risk for sustaining a deployment‐related mTBI. None of the other genotypes were associated with traumatic stress or risk for mTBI. Importantly, although prior studies report a link between BDNF and PTSD in civilians and older adult veterans that served in the Vietnam War, this is the first study to show a relationship between BDNF genotype and traumatic stress in active‐duty soldiers returning from a 12 month deployment.

Although the evidence of the contribution of the BDNF Val66Met polymorphisms to PTSD is mixed, some clinical association studies provide evidence that the BDNF genotype contributes to traumatic stress (Pivac et al. 2012; Angelucci et al. 2014). In addition, laboratory studies have begun to characterize the neurobiological mechanisms that might be implicated. For example, structural and functional brain imaging show that the Met allele accentuates attention bias to threat via amygdala–prefrontal neural circuitry (Carlson et al. 2013). Other studies report Met allele associations with hyperactivity of the amygdala (Montag et al. 2008). These findings, in conjunction with BDNF polymorphisms having strong association in the literature with synaptic plasticity (Martinowich and Lu 2008) and mood disorders (Montag et al. 2010) imply an association with increased risk of PTSD.

Combat exposure as a predictor of traumatic stress in soldiers deployed to a combat environment is not surprising due to the inherent dangers associated with war. In fact, a traumatic event is necessary for the development of PTSD and greater combat exposure has been shown to contribute to increased traumatic stress symptoms in military service members (Vasterling et al. 2010). Our data confirmed that severity of combat exposure experienced while deployed accounted for a small but significant percentage (8%) of postdeployment traumatic stress.

Mild TBI has been linked with increased risk of PTSD in deployed service members (Cooper et al. 2011; Yurgil et al. 2014). In this study, service members who sustained a deployment‐related mTBI were more likely to screen positively for traumatic stress following deployment. In addition, individuals who sustained an mTBI also had higher scores on the Combat Exposure Scale. It was not possible, however, for us to disambiguate the contributions of combat‐related psychological trauma and mTBI given the research design and our outcome measures. Those with the BDNF Met/Met genotype had a significantly higher incidence of deployment‐related mTBI (30.8%) compared to non‐Met/Met carriers (9.4%). This might be a coincidental or spurious finding. Replication of this finding is needed. It might relate to a three‐way interaction between combat exposure, mTBI, and traumatic stress. It is not known whether BDNF Met/Met status could, somehow, be associated with a lower threshold for sustaining an injury; at present, this seems unlikely.

In this study, the strongest independent predictor of postdeployment traumatic stress was predeployment traumatic stress. This is consistent with research suggesting that individuals who have experienced prior trauma are at greater risk of developing PTSD (Youngner et al. 2012). In vulnerable individuals, this increased risk appears to be partially attributed to chronic stress activation and alterations of the HPA axis, which results in hypersensitivity to actual and perceived danger (Delahanty and Nugent 2006). Measures of prior traumatic events and early childhood environment were not, however, significant independent predictors of traumatic stress at postdeployment.

Therefore, in this study it was the psychological distress itself, not the exposure to prior traumatic events, that had significant clinical implications. It is possible that the use of evidence‐based psychological treatment at predeployment with soldiers experiencing chronic traumatic stress might reduce the severity of traumatic stress experienced at postdeployment.

### Limitations

This study has several limitations. First, sample size, although sufficient to power the analyses, prevented us from doing other statistical tests to explore interactions between the different variables. This was mainly due to the disproportionately small number of homozygous Met allele carriers, which is reflected in the general population. Second, soldiers that acquire clinically apparent mental health problems associated with the combat environment, such as acute symptoms of PTSD, are screened out and referred for more rigorous behavioral health assessments and treatment, both while deployed and when returning stateside. It is likely that these avenues of medical care contributed to our relatively low incidence and low severity of traumatic stress. As such, it was necessary to use a liberal traumatic stress cutoff score on the PCL‐M (≥30), which is supported in the literature to have optimal sensitivity and specificity for screening active‐duty military service members (Bliese et al. 2008). Third, there was no longitudinal follow‐up to assess the latent trajectories of traumatic stress symptoms. Some service members have an increase in trauma symptoms months to years following deployment (Andersen et al. 2014).

### Future directions

Future studies should emphasize collecting data over longer periods of time on larger numbers of military service members in order to explore potential interactions and genetic mediators of combat‐related PTSD. In addition, the findings have clinical implications for investigating the impact of both behavioral health and gene‐targeted drug interventions with service members that are at risk for developing deployment‐related PTSD.

## Conclusion

Experiencing higher levels of traumatic stress prior to deploying to a combat environment, having the BDNF Met/Met genotype, being exposed to high levels of combat while deployed, and sustaining an mTBI while deployed independently contributed to traumatic stress following deployment. Therefore, the findings suggest that rates of PTSD in active‐duty soldiers deployed to combat environments are influenced by predeployment traumatic stress, genetics, and environmental factors.

## Disclaimer

The opinions, interpretations, conclusions, and recommendations are those of the authors and are not necessarily endorsed by the U.S. Army and/or the U.S. Department of Defense.

## Conflict of Interest

Roskamp was awarded a contract through the US Army Medical Research Acquisition Activity, US Army Medical Research and Materiel Command (USAMRMC) for assisting with data collection and analysis associated with this manuscript. G. Iverson reports personal fees from an independent practice in neuropsychology, including medical–legal work in the area of mild traumatic brain injury, with NeuroHealth Research and Rehabilitation, Inc.; grants from Canadian Institute of Health Research and INTRuST Posttraumatic Stress Disorder and Traumatic Brain Injury Clinical Consortium funded by the Department of Defense Psychological Health/Traumatic Brain Injury Research Program (X81XWH‐07‐CC‐CSDoD); and personal fees from Speaker honorariums and travel reimbursement from national and international conferences, outside the submitted work.
